# Optical, Spectroscopic, and Doppler Evaluation of “Normal” and “Abnormal” Reflexology Areas in Lumbar Vertebral Pathology: A Case Study

**DOI:** 10.1155/2012/904729

**Published:** 2012-12-17

**Authors:** Krishna Dalal, D. Elanchezhiyan, V. B. Maran, Raunak Kumar Das, Piyush Kumar, S. P. Singh, C. Murali Krishna, Jyotirmoy Chatterjee

**Affiliations:** ^1^Department of Biophysics, All India Institute of Medical Sciences, Ansarinagar, New Delhi 110 029, India; ^2^School of Medical Science and Technology, Indian Institute of Technology, West Bengal, Kharagpur 721 302, India; ^3^Chilakapati Laboratory, ACTREC, Tata Memorial Center, Kharghar, Navi Mumbai 410210, India

## Abstract

Scientific validation of reflexology requires an in-depth and noninvasive evaluation of “reflexology/reflex areas” in health and disease. The present paper reports the differential properties of “normal” and “abnormal” reflexology areas related to the lumbar vertebrae in a subject suffering from low back pain. The pathology is supported by radiological evidence. The reflexology target regions were clinically assessed with respect to colour and tenderness in response to finger pressure. Grey scale luminosity and pain intensity, as assessed by visual analogue scale scores, differentiated “normal” from “abnormal” skin. Skin swept source-optical coherence tomography recorded their structural differences. Infrared thermography revealed temperature variations. A laser Doppler study using a combined microcirculation and transcutaneous oxygen monitoring system indicated alterations in blood flow and oxygen perfusion. Raman spectroscopy showed differences in chemical signatures between these areas. The present findings may indicate a potential correlation between the reflexology areas and subsurface pathological changes, showing an association with the healthy or unhealthy status of the lumbar vertebrae.

## 1. Introduction

In the last decade, research to develop ecofriendly technological solutions for numerous problems has been intensified. The latest medical research aims at identifying diagnostic and remedial means that not only are less energy intensive and noninvasive but also have higher specificity. In this context, ancient healing practices may meet modern medical needs across many dimensions [1–3]. However, the *modus operandi* of these practices needs to be investigated scientifically to reveal their potential applications. One such practice is “reflexology,” which is a traditional, ecofriendly, therapeutic, and diagnostic practice [4]. Numerous reports indicate the therapeutic effectiveness of reflexology [5–7], and thus this study aimed to explore the fundamentals of this healing science using the latest analytical techniques. The present paper reports on the crucial features of reflexology in health and pathology, recorded through noninvasive optical, spectroscopic, and thermography techniques [8, 9].

In diagnosis and therapy, reflexology exploits potentiality of specific areas of the skin surface, called “reflexology/reflex” areas (RAs), which represent the structural and functional dimensions of various parts of the body [10]. It is assumed that the RAs provide specific cutaneous windows for noninvasive therapeutic intervention through external stimulation in diverse discrete forms and intensities [11]. In reflexology, diagnosis of a health problem is performed by visual and manual examinations of the correlating RAs on a patient's feet, hands, and/or ears, as well as by reviewing the patient's medical history. This technique suffers from nonspecificity because of its inherent heuristic nature and interobserver differences due to the lack of validated standardised parameters [12].

Skin swept-source optical coherence tomography (SS-OCT), Infrared-thermography (IR-thermography), laser Doppler imaging (using a combined microcirculation and transcutaneous oxygen monitor system), and Raman spectroscopy were used in this study for the biological characterisation of the RAs related to specific lumbar vertebrae. Optical coherence tomography is an imaging technique that uses the principle of interferometer [13]. The captured images are processed to characterise the media through which an incident laser beam traverses and interferes with its reflected ray. In SS-OCT, the tuneable wavelength of a laser source, whose wavelength rapidly sweeps within a selected wavelength band, allows the spectrum of the interferometer output to be recorded sequentially through a single detector. SS-OCT is applied in performing tomography of the skin at micron-level resolution, with a field of view of up to a few millimetres.

In IR-thermography, the thermal energy emitted from an object is transformed into the infrared band of the electromagnetic spectrum. This system is capable of producing images according to the thermal distribution of the target object. The high spatial and thermal resolving powers of the images are captured through infrared digital cameras, and the corresponding image depicts the distribution of skin temperature over the target areas. Under controlled physical conditions, these images are interpreted to detect the physiological status in response to the thermal conditions arising from body metabolism. IR-thermography has applications in medical diagnosis [14].

In laser Doppler imaging, when a laser beam irradiates skin, the back-scattered electromagnetic waves undergo Doppler shifts due to the presence of blood flow. The back-scattered ray is detected and converted into electrical signals that are processed to correlate tissue oxygenation with blood flow perfusion in the tissue [15]. It is possible to have a microvascular response in terms of the maximum dilatation capacity of the tissue with an increase in temperature under local heat provocation [16]. Local hyperaemia increases blood flow, and oxygen dissolves the lipid structure, making skin permeable to gas diffusion. The contact fluid further facilitates oxygen diffusion, which is released to the skin through the capillaries. The cutaneous oxygen tension (tcpO2) provides instant and continuous information on any impairment of the organism's ability to deliver oxygen to the tissues; that is, tcpO2 assesses tissue oxygenation [17]. This tissue oxygenation is dependent on oxygen uptake in the respiratory system, the oxygen transport/capacity of blood, and the general status of the circulatory system. The laser Doppler imaging method has many applications in diagnostic procedures for patients with ulceration from diabetic mellitus, limb ischaemia, wounds, burns, surgery, and so forth [18, 19].

Raman spectra (RS) arise from the inelastic scattering of photons from molecular vibrations [20], leading to unique spectral features that allow for their identification. Various studies have reported the efficacy of RS in finding chemical signatures in healthy and pathological conditions of the skin [21].

The multimodal evaluations carried out through this study showed for the first time differences in cutaneous (surface and subsurface) features of “normal” and “abnormal” RAs and revealed their associations with lumbar pathology supported by radiological findings of disc bulging (particularly in this paper).

## 2. Materials and Methods

### 2.1. Location and Setting of the Study

The study was conducted in a tertiary care medical institute in New Delhi, at the Indian Institute of Technology, Kharagpur, and at ACTREC, Tata Memorial Center, Navi Mumbai, India. The data were collected with the informed consent of the subject, and the ethical norms of the individual institutes were followed throughout the study.

### 2.2. Resources of the Present Study

**Figure 1:**
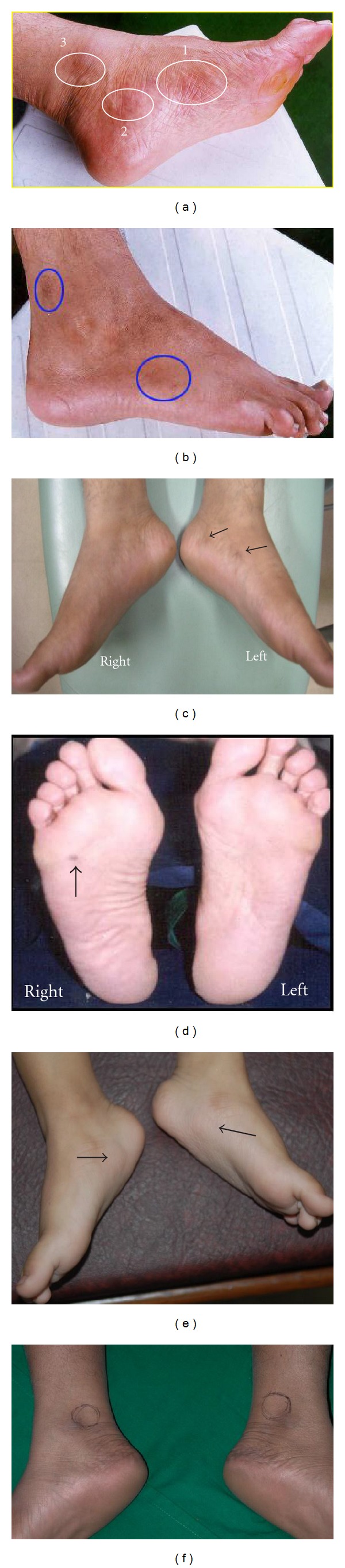
Examples of a few “abnormal” reflexology areas (RAs) with abnormal skin colour, swelling (convexity), and hollowness (concavity) formations. (a) Reddish brown colour on the RAs of pancreas (head), lumbar vertebrae, and knee-hip-sciatic nerve (medial). (b) Brown colour on the RA of knee and sciatic nerve (lateral). (c) Dark brown colour on the RAs of partial thoracic vertebrae (left) and lumbar vertebrae (left). (d) Black colour on the RA of gall bladder. (e) Swollen RAs of urinary bladder. (f) Hollowness (concavity) formation on the knee-hip-sciatic nerve (lateral).

This study was based on the previous studies by the authors and others [22, 23] on the physical features of the skin related to “normal” and “abnormal” RAs. The findings of these studies revealed that the abnormal RAs exhibited one or more of the following observables: (i) alterations in colour (reddish brown/brown/dark brown/black); (ii) a change in texture (namely, scaling/cracking skin/recurrence of corns (not due to footwear)); (iii) a formation of concavity/depression or convexity/swelling; and (iv) an increase in the local temperature of an RA. A few examples of these abnormal RAs are shown in Figure 1. Signs of tenderness in response to finger pressure on these areas were also considered abnormal [24]. Normal RAs were defined as skin regions that did not present with any of these features.

### 2.3. Study Subject

A subject of 60 years of age (female) presented with occasional pain, level 6 on a scale of 0–10 on the visual analogue scale (VAS) [25], radiating from the low back to below the knee, which occurred after standing for more than 1*⁄*2 hour. The pain was pronounced in the lower left leg and was not prominent on the right side. The patient had been suffering for a period of 6 months. In MRI studies, she was shown to have a posterior bulging disc at L4-L5 level, with a mild indentation at the thecal sac and neural foramina. Mild disc bulging was observed at L3-L4 and L5-S1, without significant neural compression. She inquired about a plausible manifestation of the pathology in the corresponding foot RAs. Other clinical status findings presented were BMI (29.2 kg/m^2^), bone density (5.3), blood pressure (average 135/75 mmHg), and glycaemic control (HbA1c = 5.6). The subject was known to suffer from hyperlipidaemia, with 25% elevated triglycerides, but she had had a well-maintained LDL/HDL ratio and serum enzyme level for the previous 10 years and was stable without pharmacological treatment. The mean values of the Toe-Brachial Index and Ankle-Brachial Index were recorded as 0.91 and 1.3, respectively, indicating that the patient did not suffer from any peripheral arterial disease [26].

### 2.4. Assessing RAs

**Figure 2:**
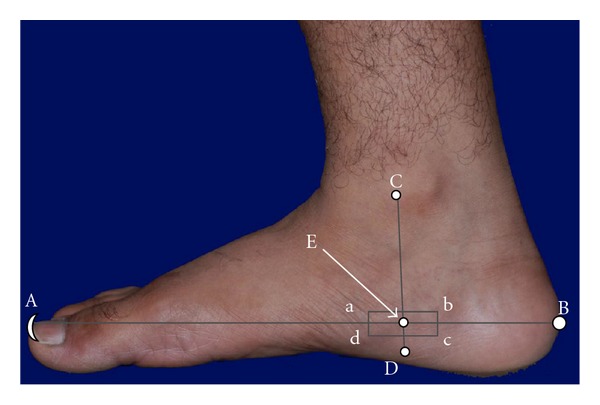
A scheme of locating the reflexology area of lumbar vertebrae on foot. Rectangle (abcd) representing the reflexology area. A: tip of great toe; B: calcaneus; C: just anterior to medial malleolus; D: below sustentaculum tali; E: centroid of abcd. AB = 23 cm and CD = 7 cm. The lengths AB and CD were considered to be standard dimensions of the referred foot.

**Figure 3:**
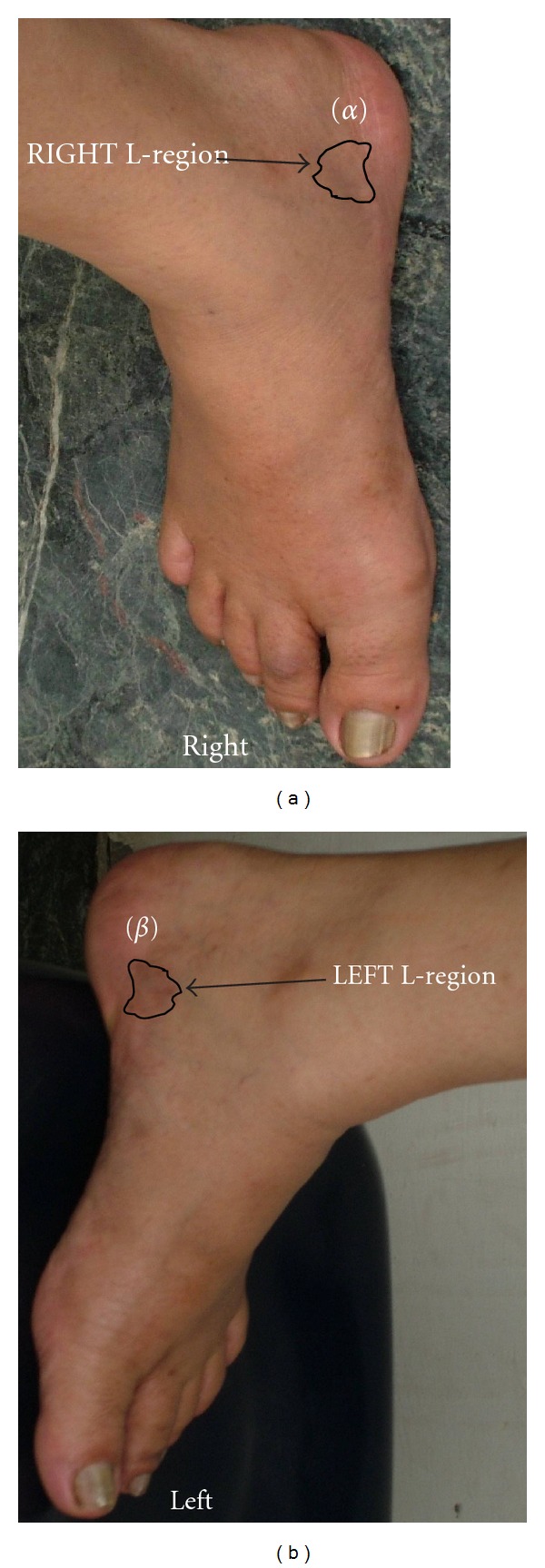
Optical image of the feet depicting “normal” reflexology area (*α*) and “abnormal” reflexology area (*β*) of the lumbar vertebrae.

In locating the lumbar RAs, the reflexology map established by Eunice Ingham D. Stophel was used [24, 27]. These RAs were charted on the bilateral medial feet. The scheme of locating the RAs is illustrated in Figure 2. For this purpose one may draw two imaginary lines (AB and CD) perpendicular to each other as shown in Figure 2. The straight line AB originates from the tip of the big toe and ends at calcaneus and the other (CD) is drawn from medial malleolus to below sustentaculum tali. The junction of these two lines formed the centroid (E) of a rectangle (abcd). The rectangle “abcd” represents RA of the lumbar vertebrae (right hand side). Identically one may draw a similar rectangle on the left foot to locate the RA of the lumbar vertebrae (left hand side). The RAs of the referred vertebrae were examined as follows. The subject was asked to clean her feet by removing any dark spots so that the feet could be viewed with optical clarity. The skin areas pertaining to the RAs of lumbar vertebrae were examined to note any difference in colour, texture, and tenderness in response to finger pressure, and the observed features were compared between the two similar areas located on the identical sites of both feet. An optical camera (Nikon D200, Japan) was used to record the skin colour of the RAs. The captured photograph is shown in Figure 3. A part of the quantised area mapped as the RA of the lumbar vertebrae on the left medial foot exhibited change in normal skin colour and this skin area was marked by LEFT L-region (Figure 3). The corresponding skin area on the right foot with normal skin colour was marked by RIGHT L-region (Figure 3). LEFT L-region exhibited dark brown colour and RIGHT L-region did not show any change in colour. The grey scale luminosity (without a unit, in accordance with the specification of the supplier) of these two regions was measured by Axiovision software (version 4.7.2, Carl Zeiss, Germany). Moderate finger pressure (~35 N/cm^2^, assessed by a pedography system, emed-at/2, Novel gmbh, Germany [28]) was applied to assess the tenderness of these RAs. The intensity of pain was measured by using a visual analogue scale (VAS) in the range from “0” to “10,” with “0” indicating “no pain” and “10” indicating “excruciating pain.” The physical observations on change in colour (dark brown) and tenderness (VAS score 6) led to designate the LEFT L-region of the medial foot (Figure 3) as “abnormal” RA and the skin area with the absence of colour change and tenderness of VAS score 1 on the RIGHT L-region of the medial foot (Figure 3) was identified as “normal” RA.

### 2.5. Swept Source-OCT (SS-OCT) Technique

The SS-OCT is an appropriate choice in tomography imaging of cutaneous tissues with micron-level resolution. The SS-OCT (OCM1300SS, Thorlabs Incorporated, Newton, NJ) incorporates a high-speed frequency-swept external cavity laser (*λ*_central_ = 1325 nm) with 3-dB spectral bandwidth (>100 nm) and an average output power of 10 mW. The SS-OCT system houses a Michelson interferometer and a built-in Mach-Zehnder interferometer (MZI, Thorlabs INT-MZI-1300) to provide a frequency clock of the laser. The output laser of the Mach-Zehnder interferometer is coupled with a Michelson interferometer to split into the sample and reference paths by a broadband coupler (Thorlabs, FC1310-70-50-APC). In practice, light is focused onto the specific areas by a long distance objective, while maintaining a clearance (>25 mm) between optics. The beam (width = 660 nm) is coupled with the study arm to locate the scanning trace of the laser. This OCT system can produce high-resolution cross-sectional images for different cutaneous layers, with axial and transverse resolutions of 9 *μ*m and 15 *μ*m, respectively (data supplied by the manufacturer). In this system, the axial scans (A-scans) of the laser were performed at a sweeping frequency of 16 kHz to construct the depth profile (~2 mm). The details of the instrumentation are described elsewhere [29]. OCT imaging (with a 3 mm imaging width) was performed at LEFT L-region (“abnormal” RA) and RIGHT L-region (“normal” RA). The images were taken digitally at 512 × 512 pixels and analysed by converting them into the grey scale using Adobe Photoshop 7.0. The optical intensity distribution and thickness of the skin layers were measured by the Axiovision software (version 4.7.2, Carl Zeiss, Germany). To consider the ground truth of the images, particularly the thickness of the cutaneous layers, the related domain knowledge expert was consulted.

### 2.6. IR-Thermography Method

The thermography camera had an LWIR 7.5–14 *μ*m uncooled FPA detector. The visible image of the IR-thermography (VarioCAM hr Research 780, Infratec, Germany) was with a frame of 1280 × 960 pixels with a thermal resolution of 0.03°C (±1.5%). The field of view was 30 × 23°, with a 1.0 f-number and a dynamic range of 16 bit. The radiometric video transfer, recording, and analysis were conducted using IRBIS Professional (Germany) software. Perfect image merging of a 1.3 Megapixel Infrared image with a 1.3 Megapixel visual image assisted in selecting the areas. Prior to the IR-thermography, the subject was instructed to refrain from using any oily substance and detergent for 1 day and to rest for 15 minutes before the examination. While capturing the IR signals, the distance (*D*) between the IR detector and the target skin was between 95 cm and 100 cm.

### 2.7. Laser Doppler Imaging Technique (Using a Combined Microcirculation and Transcutaneous Oxygen Monitoring System)

This study utilised a combined microcirculation and transcutaneous oxygen monitoring system with a Periflux 5000 microcirculation examiner (Perimed, Sweden) to collect the following data on the peripheral circulation of the skin at RAs: (i) tissue viability, which was assessed by a blood flow perfusion scan, and (ii) tissue oxygenation, as assessed by transcutaneous oxygen partial pressure (tcpO_2_) [30]. For this purpose, a time period of 20 minutes was allotted for each examination. Microcirculatory heat responses at the same positions were recorded in terms of blood perfusion units by elevating the localised skin temperature from 29°C to 44°C with cutaneous electrodes. The change in tcpO_2_ over time during the release of arm pressure from 136 mmHg to atmospheric pressure was measured by capturing the electrical signals. The signals were converted into a tcpO_2_ reading in mmHg, resulting in the measurement of the transcutaneous partial tension of the local oxygen level in the tissue.

### 2.8. Raman Spectroscopy Technique

In this study, regions on both “normal” and “abnormal” RAs were fixed, and a total of 20 spectra (12 in “normal” RA and 8 in “abnormal” RA) were recorded using commercially available HE-785 Raman Spectrometer (Jobin-Yvon-Horiba, France). Briefly, this system consists of a diode laser (*λ*_ex_-785 nm) as an excitation source and a CCD (Synapse)-coupled with High Efficiency (HE) spectrograph as the dispersion and detection element. The instrument has no movable parts and spectral resolution, as per the manufacturers specification was ~4 cm^−1^. Commercially available In Photonics (Inc, Downy St. USA) probe consisting of 105 *μ*m excitation fiber and 200 *μ*m collection fiber (NA-0.40) was used to couple excitation source and detection system. As per specifications of manufacturer of the InPhotonics probe, theoretical spot size and depth of field are 105 *μ*m and 1 mm, respectively. Spectral acquisition parameters were *λ*_ex_-785 nm and laser power-100 mW; spectra were integrated for 10 seconds and averaged over 3 accumulations. During pre-processing, spectra were corrected for the CCD response and for interfering fibre signals. First derivative spectra in the 900–1800 cm^−1^ region were used as input for Principal Component Analysis (PCA) using in-house MATLAB-based software [31].

### 2.9. Analyses of Data

The observations recorded from the clinical photographs on RAs, SS-OCT, and IR-thermography images were statistically evaluated by a two-sample *t*-test with a 95% confidence interval using SPSS statistics 17.0 software (SPSS, Inc., USA). The variations in the diameters of the vessels were presented by box-and-whisker plots [32]. Raman spectroscopy findings were evaluated by PCA. The % changes in the blood perfusion unit and tcpO_2_ were calculated using the following:

(1) $$\begin{array}{c} \%\;\;\text{change}=\frac{{\text{parameter}}_{“\text{normal}"}-{\text{parameter}}_{“\text{abnormal}"}}{{\text{parameter}}_{“\text{normal}"}}\times 100. \end{array}$$

Parameter represents the blood perfusion unit or tcpO_2_ (mm of Hg). The parameter_*“*normal"_ and parameter_*“*abnormal"_ stand for the values of these two items at “normal” RA (RIGHT L-region of Figure 3) and “abnormal” RA (LEFT L-region of Figure 3), respectively.

## 3. Results and Discussion

The present case study did not attempt to correlate low back pain with intervertebral disc bulging but presented the radiological information to show evidence of disc abnormality. This is the first time that tomographic and spectroscopic observations in RAs have been reported to describe the differences in “normal” and “abnormal” conditions in a subject with low back pain radiating below the knee. The subject, who had suffered from low back pain for 6 months, would be expected to manifest characteristics of inflammation [33]. This manifestation might have some degree of an anatomical bias that could be correlated with RA changes. In this context, the analytical techniques used in this study are valuable for depicting differences in the structural and functional status of the RAs noninvasively with respect to their surface and subsurface features. The depth of penetration of the laser beams used for SS-OCT and Raman spectroscopy was approximately ~1.75 mm, as mentioned by the suppliers.

### 3.1. Optical Images and Tenderness of RAs

Figures 3(*α*) and 3(*β*), representing “normal” RA (RIGHT L-region) and “abnormal” RA (LEFT L-region), respectively, depict the statistically significant difference in the grey scale luminosity of the RAs in the clinical photographs between these RAs (*P* value < 0.01) (Table 1). Table 1 further depicts the differential response in terms of tenderness under defined finger pressure.

### 3.2. SS-OCT Findings

**Figure 4:**
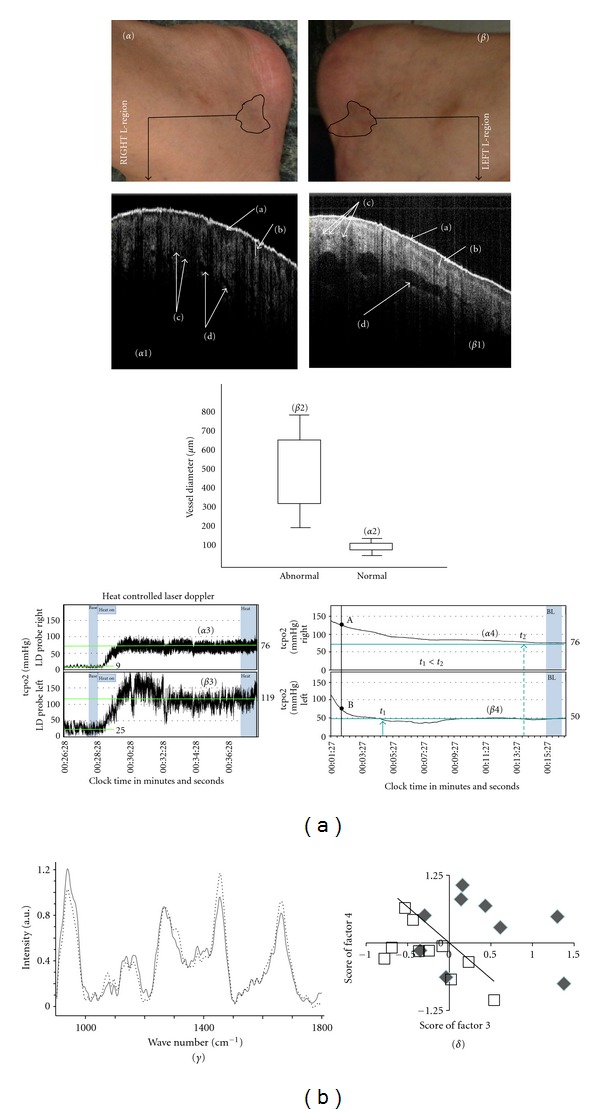
Multimodal images of reflexology areas (RAs) of lumbar vertebrae and the corresponding analytical results. (*α*) and (*β*) represent “normal” RA and “abnormal” RA, respectively, (cut section of Figure 3). (*α*1) and (*β*1) represent the OCT images of “normal” and “abnormal” RAs, respectively, with (a) stratum corneum; (b) viable epidermis; (c) microvessels; (d) macrovessels. (*α*2) and (*β*2) depict box-and-whisker plots for the diameters of macrovessels in “normal” and “abnormal” RAs, respectively. (*α*3) and (*β*3) represent spectra for blood flow perfusion in “normal” and “abnormal” RAs, respectively. (*α*4) and (*β*4) represent the time variation of transcutaneous oxygen tension under arm pressure release in “normal” and “abnormal” RAs, respectively. (*γ*) depicts the mean Raman spectra for “normal” (solid line) and “abnormal” (dotted lines) in the range of 900–1800 cm^−1^. (*δ*) shows PCA clustering of “abnormal” (hollow squares) and “normal” (solid diamonds) molecular signatures of Raman spectra in the range of 900–1800 cm^−1^.

SS-OCT was able to detect the surface and subsurface skin structure changes of the RAs in pathological conditions, as depicted in Figures 4(*α*1), 4(*β*1), 4(*α*2), and 4(*β*2) and Table 1. Optical tomographs revealed differences between “abnormal” and “normal” RAs in terms of the lucidity and thickness of the viable epidermis and the diameters of the vessels. In the viable epidermis (VEpi) of the skin representing “abnormal” RA, lucidity increased, thickness decreased, and the diameters of blood vessels increased significantly (*P* < 0.01). These changes, especially the elongated diameters of blood vessels, could corroborate the increased tenderness of “abnormal” RA.

### 3.3. IR-Thermography Findings

The IR-thermography recorded that the temperature of “abnormal” RA was 0.98°C, higher than that of “normal” RA (Table 1). This finding corroborates the clinical observations and the SS-OCT findings in the context of plausible inflammation with low back pain (Sections 3.1 and 3.2).

### 3.4. Laser Doppler Imaging Technique (Using a Combined Microcirculation and Transcutaneous Oxygen Monitoring System)

The microcirculation and transcutaneous oxygen monitoring system recorded 177.7% and 51% elevation in blood perfusion units at baseline and under heat provocation, respectively, in “abnormal” RA compared with those of “normal” RA. A 34% reduction in cutaneous oxygen partial pressure was observed in the “abnormal” site. The data are presented in Table 1, and the nature of the variations is shown in Figures 4(*α*3), 4(*β*3), 4(*α*4), and 4(*β*4), respectively. This characteristic decrease in oxygen partial pressure and increase in blood perfusion indicates a type of stagnation under an inflammatory condition. These findings corroborate previous findings, as presented in Sections 3.2 and 3.3. It was observed that changes in tcpO_2_ over time during the release of arm pressure (from 136 mmHg to atmospheric pressure) manifested a sharp fall in the midst of a falling trail in “abnormal” RA, with a slope of (−4.3) at point B of Figure 4(*β*4), whereas there was a slow and steady decrease for “normal” RA (slope −1.0, at point A of Figure 4(*α*4)). These results may be due to the presence of the larger size of the macrovessels that were filled with blood/fluid in “abnormal” RA, as detected by SS-OCT (presented in Section 3.2 and in Figure 4(*β*2)). Thus, the time for the elevated partial oxygen pressure to reach the steady state at “normal” area was more than that at the “abnormal” area (*t*_2_ ≫ *t*_1_ in Figures 4(*α*4) and 4(*β*4)). This reduced time for “abnormal” RA may be the manifestation of the functional abnormality of the vascular structure of this cutaneous area.

### 3.5. Raman Spectroscopy Findings

The baseline corrected mean spectra for “normal” (solid line) and “abnormal” (dotted line) RAs are shown in Figure 4(*γ*). Major bands from the contribution of amide I (around 1660 cm^−1^) and amide III (around 1260 cm^−1^) and ester bands from lipids (around 1745 cm^−1^) were observed. The band around 1450 cm^−1^ represents vibrations from *δ*CH2, while the bands at 1380 cm^−1^ and 1580 cm^−1^ are reported as signatures of melanin. According to the literature, these two bands can be observed in both natural melanin and synthetic eumelanins and are prone to minor environment related variations [21]. These can be assigned to in-plane stretching of the aromatic ring and linear stretching of the C–C bonds within the ring, with some contributions from the C-H vibrations in the methyl and methylene groups. Major differences were observed in the amide III, *δ*CH2 stretch and amide I regions. To understand variations and similarities across the spectra of both “normal” and “abnormal” RAs and to explore the feasibility classifications, unsupervised PCA was performed. The scatter plot obtained after PCA is shown in Figure 4(*δ*). Separate clusters belonging to “normal” and “abnormal” RAs, indicating changes in the bio-molecular compositions of these areas, were obtained.

### 3.6. Overall Observations and Outcome

Observations from the data recorded through the multimodal techniques may be collectively stated. Features observed in the SS-OCT images and data recorded in a combined microcirculation and transcutaneous oxygen monitoring system indicated that “abnormal” RA exhibited the subsurface attributes of inflammation. The comparative smoothness of the stratum corneum and enhanced diameters of blood vessels indicated stretching of the skin and dilatation of the vessels in an inflammatory process. Further enhanced blood perfusion [34] at baseline and increased perfusion under heat provocation corroborated vessel dilatation with a concomitant increase of vessel porosity [35]. The decrease in oxygen partial pressure supports the fact that an abnormal RA would be in an inflammatory condition due to hypoxia [36, 37]. The elevation in localised temperature, as measured by the IR-thermography of “abnormal” RA, further supports the finding of an inflammatory state in the referred skin area [38] and an inflamed state of skin induces hyperalgesia [39].

## 4. Conclusion

The multimodal evaluations of the target cutaneous and subcutaneous regions showed differences in the characteristics of “normal” and “abnormal” reflexology areas in a patient with low back pain. The clinical, structural, molecular, thermal, and functional differences between these two areas could be identified, and the findings were indicative of the inflammatory changes. The findings of the present study, if extended to more subjects, may aid in the development of an evidence-based technique to correlate skin changes in lumbar pathology.

## Figures and Tables

**Table 1:** Multimodal evaluation of  “normal” and “abnormal” reflexology areas for lumbar vertebrae.

Technique	Feature	Reflexology areas		*P*-value/remarks
		“Normal”	“Abnormal”	
Clinical photography	Grey scale luminosity	158.1 ± 12.0	105.9 ± 13.9	<0.001

VAS scoring of tenderness under defined finger pressure (~35 N/cm^2^)	Tenderness (VAS score)	1	6	Increased tenderness in “abnormal” RA

SS-OCT	Viable epidermis thickness (*μ*m) mean ± SD	329.9 ± 13.5	200.0 ± 35.8	<0.001
	Viable epidermis luminosity	59.8 ± 28.2	84.0 ± 28.9	<0.001

IR-thermography	Temperature (°C) mean ± SD	34.58 ± 0.16	35.44 ± 0.05	<0.001

	Blood perfusion unit at base line	9	25	177.7% blood perfusion enhanced in “abnormal” RA
Combined microcirculation and transcutaneous oxygen monitor system	Blood perfusion unit under heat provocation	76	119	51.0% blood perfusion elevated in “abnormal” RA
	Oxygen partial pressure (mm of Hg) under stable condition	76	50	34.0% oxygen partial pressure reduced in “abnormal” RA

## References

[1] Mao-liang C (1993). *Chinese Acupuncture and Moxibustion*.

[2] Beresford-Cooke C (2003). *Shiatsu Theory and Practice: A Comprehensive Text for the Student and Professional*.

[3] Gregor M (2007). *Ashtanga Yoga, Practice and Philosophy*.

[4] Tiran D, Chummun H (2005). The physiological basis of reflexology and its use as a potential diagnostic tool. *Complementary Therapies in Clinical Practice*.

[5] Hughes CM, Smyth S, Lowe-Strong AS (2009). Reflexology for the treatment of pain in people with multiple sclerosis: a double-blind randomised sham-controlled clinical trial. *Multiple Sclerosis*.

[6] Gunnarsdottir TJ, Peden-McAlpine C (2010). Effects of reflexology on fibromyalgia symptoms: a multiple case study. *Complementary Therapies in Clinical Practice*.

[7] Lee YM (2011). Effects of self-foot reflexology on stress, fatigue, skin temperature and immune response in female undergraduate students. *Journal of Korean Academy of Nursing*.

[8] Zhao Y, Chen Z, Saxer C, Xiang S, de Boer JF, Nelson JS (2000). Phase-resolved optical coherence tomography and optical Doppler tomography for imaging blood flow in human skin with fast scanning speed and high velocity sensitivity. *Optics Letters*.

[9] Tuchin VV (2002). *Handbook of Optical Biomedical Diagnostics*.

[10] Mackereth PS, Tiran D (2002). *Clinical Reflexology: A Guide for Health Professionals*.

[11] Louise K (2009). *The Reflexology Bible, the Definitive Guide to Pressure Point Healing*.

[12] Ernst E, Posadzki P, Lee MS (2011). Reflexology: an update of a systematic review of randomised clinical trials. *Maturitas*.

[13] Galle MA, Saini SS, Mohammed WS (2012). Chromatic dispersion measurements using a virtually referenced interferometer. *Optics Letter*.

[14] Jones BF (1998). A reappraisal of the use of infrared thermal image analysis in medicine. *IEEE Transactions on Medical Imaging*.

[15] Schilling MF, Watkins AE, Watkins W (2002). Is human height bimodal?. *American Statistician*.

[16] Minson CT (2010). Thermal provocation to evaluate microvascular reactivity in human skin. *Journal of Applied Physiology*.

[17] Carreau A, Hafny-Rahbi BE, Matejuk A, Grillon C, Kieda C (2011). Why is the partial oxygen pressure of human tissues a crucial parameter? Small molecules and hypoxia. *Journal of Cellular and Molecular Medicine*.

[18] Isner JM, Pieczek A, Schainfeld R (1996). Clinical evidence of angiogenesis after arterial gene transfer of phVEGF165 in patient with ischaemic limb. *The Lancet*.

[19] Wilson SB, Jennings PE, Belch JJF (1992). Detection of microvascular impairment in type I diabetics by laser Doppler flowmetry. *Clinical Physiology*.

[20] Raman CV, Krishnan KS (1928). A new type of secondary radiation. *Nature*.

[21] Huang Z, Lui H, Chen XK, Alajlan A, McLean DI, Zeng H (2004). Raman spectroscopy of in vivo cutaneous melanin. *Journal of Biomedical Optics*.

[22] Jones S (2009). *Simply Reflexology*.

[23] Last W Reflexology. http://www.health-science-spirit.com/reflexology.html.

[24] Byers DC (1987). *Better Health with Foot Reflexology*.

[25] Sahni N, Anand LK, Gombar K (2011). Effect of intra-operative depth of anesthesia on postoperative pain and analgesic requirement: a randomized prospective observer blinded study. *Journal of Anaesthesiology Clinical Pharmacology*.

[26] Shi P, Hu S, Zhut Y, Zheng J, Qiu Y, Cheang PYS (2009). Insight into the dicrotic notch in photoplethysmographic pulses from the finger tip of young adults. *Journal of Medical Engineering and Technology*.

[27] Bahl IP (2005). *Listen to Your Feet*.

[28] Filippin NT, Bacarin TDA, da Costa PHL (2008). Comparison of static footprints and pedobarography in obese and non-obese children. *Foot and Ankle International*.

[29] Barui A, Banerjee P, Patra R (2011). Swept-source optical coherence tomography of lower limb wound healing with histopathological correlation. *Journal of Biomedical Optics*.

[30] Wang CJ, Wu RW, Yang YJ (2011). Treatment of diabetic foot ulcers: a comparative study of extracorporeal shockwave therapy and hyperbaric oxygen therapy. *Diabetes Research and Clinical Practice*.

[31] Ghanate AD, Kothiwale S, Singh SP, Bertrand D, Krishna CM (2011). Comparative evaluation of spectroscopic models using different multivariate statistical tools in a multicancer scenario. *Journal of Biomedical Optics*.

[32] Pupovac V, Petrovecki M (2011). Summarizing and presenting numerical data. *Biochemia Medica*.

[33] Roy RA, Boucher JP, Comtois AS (2010). Inflammatory response following a short-term course of chiropractic treatment in subjects with and without chronic low back pain. *Journal of Chiropractic Medicine*.

[34] Kuo YR, Wu WS, Hsieh YL (2007). Extracorporeal shock wave enhanced extended skin flap tissue survival via increase of topical blood perfusion and associated with suppression of tissue pro-inflammation. *Journal of Surgical Research*.

[35] Bezuidenhout D, Davies N, Zilla P (2002). Effect of well defined dodecahedral porosity on inflammation and angiogenesis. *ASAIO Journal*.

[36] Moreau C, Devos D, Brunaud-Danel V (2005). Elevated IL-6 and TNF-*α* levels in patients with ALS: inflammation or hypoxia?. *Neurology*.

[37] Karhausen J, Haase VH, Colgan SP (2005). Inflammatory hypoxia: role of hypoxia-inducible factor. *Cell Cycle*.

[38] Page EH, Shear NH (1988). Temperature-dependent skin disorders. *Journal of the American Academy of Dermatology*.

[39] Kilo S, Schmelz M, Koltzenburg M, Handwerker HO (1994). Different patterns of hyperalgesia induced by experimental inflammation in human skin. *Brain*.

